# Duplex Molecular Assay Intended for Point-of-Care Diagnosis of Influenza A/B Virus Infection

**DOI:** 10.1128/JCM.00740-13

**Published:** 2013-09

**Authors:** Liang-Ta Wu, Isabelle Thomas, Martin D. Curran, Joanna S. Ellis, Surendra Parmar, Neha Goel, Pia I. Sharma, Jean-Pierre Allain, Helen H. Lee

**Affiliations:** Diagnostics Development Unit, Department of Haematology, University of Cambridge, Cambridge, United Kingdoma; Scientific Institute of Public Health, Brussels, Belgiumb; Clinical Microbiology and Public Health Laboratory, Health Protection Agency, Addenbrooke's Hospital, Cambridge, United Kingdomc; Virus Reference Department, Microbiology Services, Public Health England, Colindale, London, United Kingdomd; Department of Haematology, University of Cambridge, Cambridge, United Kingdome

## Abstract

Early diagnosis and management of influenza virus infection directly correlates with the effectiveness in disease control. Current molecular influenza virus tests were designed for use in diagnostic testing facilities, where sophisticated equipment and highly trained technicians are available. A longer turnaround time for the centralized testing than when testing near the sample source could delay the initiation of medical intervention, thereby reducing the efficacy of antiviral treatment. The new assay, the SAMBA (simple amplification-based assay) Flu duplex test, is a dipstick-based molecular assay developed to provide a simple, accurate, and cost-effective solution for the diagnosis of influenza A/B viruses intended for near-patient testing. The test presents an alternative format of influenza virus molecular testing that utilizes isothermal amplification and visual detection of nucleic acid on a test strip. The entire test procedure (extraction, amplification, and detection) is integrated into an enclosed semiautomated system. Analytically, the SAMBA Flu duplex test detects 95 and 85 copies of viral genomes for influenza A and B viruses, respectively, with no cross-reactivity observed against other common respiratory pathogens. The clinical performance was established by blind testing of 328 nasal/throat and nasopharyngeal swab specimens from the United Kingdom and Belgium and comparing the results with the quantitative reverse transcription-PCR method routinely used in two public health laboratories. The SAMBA Flu duplex test showed a clinical sensitivity and specificity of 100% and 97.9% for influenza virus A and 100% and 100% for influenza virus B. The test provides a new technology that could facilitate simple and timely identification of influenza virus infection, potentially resulting in more efficient control measures.

## INTRODUCTION

Influenza viruses are major human pathogens that cause a significant number of illnesses and deaths each year during the seasonal epidemic. In the United States alone, seasonal epidemic influenza has been estimated to result in 31 million outpatient visits and an annual total economic burden of $87 billion (1, 2). The elderly, children, and individuals with underlying medical conditions are at risk of increased morbidity and mortality caused by influenza virus infection (3, 4). Furthermore, emerging influenza virus strains have the potential of sustaining efficient human-to-human transmission and causing a global pandemic, such as the recent outbreak of influenza A(H1N1)pdm09 (5). In terms of treatment options, neuraminidase inhibitors (NIs) are currently the first-line influenza virus antiviral drugs. NI administration is recommended during the early phase of the disease (between 24 and 72 h after illness onset), when the replication of influenza virus peaks (6). At a community level, timely implementation of patient isolation and social distancing measures, including school closures, have been shown to reduce viral transmission (7, 8). The control of influenza virus infection highlights the critical role of an efficient diagnostic assay that enables prompt identification of an infected person and the initiation of subsequent disease management.

At present, there is a wide range of techniques available to achieve different levels of diagnosis, including serology, conventional virus culture, direct fluorescent antibody (DFA) test, rapid immunoassay (rapid test), and nucleic acid test (NAT). Serology and virus culture involve lengthy laboratory processes (2 to 14 days) that render these methods unsuitable for rapid clinical diagnosis (9, 10). R-mix cultures have been widely used for the detection of influenza viruses, with an improved turnaround time of 1 to 2 days (11, 12). DFA, although it significantly reduces the turnaround time to 2 to 6 h, is generally less sensitive than virus culture and requires trained personnel to perform and interpret results (13, 14). Rapid tests for influenza virus based on viral antigen detection by lateral flow immunochromatographic dipstick assay have become widely used in point-of-care settings, such as emergency rooms, doctors' clinics, etc. Rapid tests are user-friendly and can be performed with minimal training without the requirement of complex equipment. In terms of turnaround time, rapid tests confer the highest efficiency, with the results known within 10 to 15 min. However, it has been shown that rapid tests exhibit inconsistent test performance, especially in the detection of A(H1N1)pdm09 viruses (15–18). Due to the limitations of the rapid immunoassays and the increasing availability of molecular technologies, a number of molecular assays for in-house research and diagnostic use have become available (14, 19). The majority of the molecular tests are based on real-time quantitative reverse transcription-PCR (qRT-PCR) and require trained operators and specialized equipment for the extraction and amplification steps (20–23). In addition, the cost and unavailability of centralized testing pose a significant threat to the control of influenza virus in resource-limited settings.

Here we describe a new molecular assay for the detection influenza A and B virus nucleic acids that was developed on the SAMBA (simple amplification-based assay) molecular platform to achieve a high level of performance while providing a fast and easy-to-use diagnostic solution (24). The test, the SAMBA Flu duplex test, couples isothermal amplification with visual detection of nucleic acid on the dipstick to allow a simple and sensitive diagnosis of influenza virus A and B infections.

## MATERIALS AND METHODS

### Control virus cultures.

Influenza viruses A/Brisbane/59/2007 (former seasonal H1N1), A/Brisbane/10/2007 (H3N2), B/Florida/4/2006, B/Malaysia/2506/2004, A/Wisconsin/67/2005 (H3N2), and A/England/195/2009 [A(H1N1)pdm09] were provided by Public Health England (PHE) Colindale. These viruses were cultured in the allantoic cavities of embryonated hen eggs, quantified by plaque assay, heat inactivated (56°C for 90 min), and diluted in virus transport medium (VTM; manufactured at PHE) to which 10^6^/ml Hep2 cells were added to simulate a clinical sample. A/California/7/2009 [A(H1N1)pdm09], A/Perth/16/2009 (H3N2), and B/Brisbane/60/2008, as well as other cultured viruses (used in determining analytical reactivities) from The National Institute for Biological Standards and Control (NIBSC) were received in a freeze-dried format and reconstituted in 250 μl of H_2_O followed by heat inactivation and quantification with the in-house qRT-PCR system using *in vitro* transcript as the standard (details are described below). Inactivated cultures were diluted in pooled negative swab samples in Copan universal transport medium (UTM; Copan, Brescia, Italy) to the desirable concentration (the number of copies/test [in 250 μl of sample]) before RNA extraction. A panel of 10 respiratory viruses was purchased from Qnostics (Glasgow, United Kingdom). Escherichia coli bacteriophage MS2 ATCC 15597-B1 was acquired from ATCC and propagated and titrated according to the supplier's instructions.

### Clinical samples.

A total of 328 samples were collected from two institutions (described below) for the clinical evaluation. The samples were all surplus samples left from routine respiratory testing. The clinical results and patient details were initially blinded to the operators of the SAMBA Flu duplex test. These samples were in two groups: (i) 41 prospectively collected mixed nasal/throat swab specimens collected between 7 March and 5 April 2012 from the Clinical Microbiology and Public Health Laboratory, Health Protection Agency (HPA), Addenbrooke's Hospital, Cambridge, United Kingdom, and (ii) 287 archived nasopharyngeal swab specimens provided by the Scientific Institute of Public Health (WIV-ISP), Brussels, Belgium. These samples were submitted to WIV-ISP for testing during and between influenza seasons from 2010 to 2012. Out of these 219 positive archived samples (124 influenza A virus positive and 95 influenza B virus positive), 8 had threshold cycles (*C_T_*) of ≥35 (3.7%), 30 had a *C_T_* between 30 and <35 (13.7%), 72 had a *C_T_* of 25 to <30 (32.9%), 99 had a *C_T_* of 20 to <25 (45.2%), and 10 had a *C_T_* of <20 (4.6%) (*C_T_* values were provided by WIV-ISP after SAMBA testing). For both groups (from the United Kingdom and from Brussels), screening and confirmatory data were retrospectively available.

### RNA preparation for quantification.

For quantification of the culture stock, the diluted virus in UTM (1 in 100 dilution) was extracted using a QIAamp viral RNA minikit (Qiagen, Crawley, United Kingdom) according to the manufacturer's instructions (80-μl elution volume).

### Production of *in vitro* transcript as quantification standard.

RNA transcripts containing the influenza A/B virus conserved sequences targeted by SAMBA were produced for use in the quantification of cultured viruses. In summary, cDNAs of A/Brisbane/59/2007 and B/Florida/4/2006 were generated from RNA extract by using the SuperScript III first-strand synthesis system for RT-PCR (Invitrogen, Paisley, United Kingdom). Amplicons of influenza A/B virus sequences chosen as targets for the SAMBA Flu duplex test were amplified by primer sets (for influenza A virus, A.M-F, 5′-GCATTITGGACAAAICGTCTAC-3′, and A.M-R, 5′-CTAAAGACAAGACCAATICTGTCA-3′; for influenza B virus, B.NS-F, 5′-CATCTTCTTCATCCTCCACTGTAA-3′ and B.NS-R, 5′-GGATACAAGTCCTTATCAACTCTGC-3′) flanking the conserved regions using Hot Master *Taq* polymerase (5 Prime). The 113-bp (influenza A virus) and 131-bp (influenza B virus) amplicons were purified by using a QIAquick PCR purification kit (Qiagen) followed by cloning into the pCRII vector using a TA cloning kit dual promoter (with the pCRII vector; Invitrogen). Influenza A/B virus plasmids were transformed into One Shot TOP 10F′ chemically competent E. coli cells (Invitrogen) and purified by using a QIAprep Spin miniprep kit (Qiagen) before the orientation of insert was confirmed by sequencing with the primers M13 forward (5′-TGTAAAACGACGGCCAGT-3′) and M13 reverse (5′-CAGGAAACAGCTATGAC-3′). Plasmids with the correct insert sequence were selected to produce PCR amplicons by using M13 forward and reverse primers followed by PCR purification. The purified amplicons were quantified using the NanoDrop ND-1000 system (Thermo Scientific), and the recommended amount of amplicons was transcribed *in vitro* with a MEGAshortscript T7 kit (influenza A virus) or MEGAscript SP6 kit (influenza B virus; Ambion, Paisley, United Kingdom). The transcripts were purified using a MEGAclear kit (Ambion), and their concentrations (in ng/μl) were determined by using the NanoDrop system. Finally, the number of copies per microliter for each preparation of transcript was calculated according to the following formula: RNA amount (in copy/μl) = [(RNA concentration, in ng/μl) × 10^−9^/molecular weight of RNA (in g/mol)] × (6.022 × 10^23^ copies/mole). Influenza A/B virus transcripts were diluted in water in 10-fold serial dilutions from 10^6^ to 10^1^ copies/5 μl to be used as standards in the qRT-PCR.

### In-house real-time RT-PCR.

An in-house one-step real-time RT-PCR (qRT-PCR) assay was developed for the quantification of the influenza virus cultures (not intended to be used as a diagnostic assay), using RNA transcripts as the standards. The primers (Eurofins MWG Operon, Ebersberg, Germany) used were the following: influenza A forward, 5′-GACCRATYYTGTCACCTCTGAC-3′, influenza A reverse, 5′-AGGGCATTYTGGACAAAKCGTCTA-3′, influenza B forward, 5′-TAYAAGTCCTTATYAACTCTGCATA-3′, and influenza B reverse, 5′-CATCTTCTTCATCCTCCACTGTAA-3′. The probes (Life Technologies, Warrington, United Kingdom) used were influenza A probe, 6-carboxyfluorescein (FAM)–5′-ATTTGTGTTCACGCTCACCG-3′–MGBNFQ (MGBNFQ is the minor-groove binder nonfluorescent quencher) and influenza B probe, VIC-5′-CATATGACCAGAGTGGAAGG-3′-MGBNFQ. The qRT-PCR was performed using a SuperScript III Platinum one-step qRT-PCR kit (Invitrogen) on an Mx3005p QPCR system (Agilent Technologies, Stockport, United Kingdom). The qRT-PCR mixture was prepared in a volume of 25 μl containing 0.8 μM influenza virus A forward and reverse primers, 0.6 μM influenza virus B forward and reverse primers, 0.2 μM influenza virus A probe, 0.15 μM influenza virus B probe, 4% dimethyl sulfoxide, and 0.7 μl of SuperScript III RT/Platinum *Taq* mix. The cycling conditions started with an initial incubation at 50°C for 30 min and 95°C for 2 min, followed by 45 cycles of denaturation at 95°C for 15 s and an extension step at 60°C for 1 min. The fluorescence signal was acquired at the end of each cycle. This qRT-PCR chemistry was optimized by using transcript produced as described in the previous section. In terms of test performance, this method consistently detected 10 copies of transcript standards per reaction mixture. For quantification of cultured virus, 5 μl of RNA extract was amplified in duplicate (at least two extractions were performed) with the influenza A/B virus transcript standards, and the titer of the stock virus was calculated, taking into account the amplification, elution, and extraction volumes.

### Detection of influenza A/B virus by two reference methods.

Clinical specimens collected from two sites (HPA Cambridge and WIV-ISP) were screened independently by qualified biomedical scientists for the presence of influenza virus according to their routine testing protocols.

### (i) Detection and typing of influenza A and B viruses at HPA Cambridge.

Clinical specimens were screened for influenza virus with qRT-PCR assays at the regional clinical microbiology laboratory in the Cambridge Health District, United Kingdom. Our previously reported generic quadriplex assay (25, 26) was upgraded for this study to a pentaplex assay capable of detecting all influenza A virus subtypes, influenza B virus, the hemagglutinin A(H1)pdm09 and H3 subtypes, and bacteriophage MS2, and it was performed essentially as outlined in reference 25, with the following modifications. The hemagglutinin H5 subtype primers and probe were replaced with A(H1)pdm09 hemagglutinin-specific primers and probe, H1F, 5′-TCAACAGACACTGTAGACACAGTACT-3′; H1R, 5′-GTTTCCCGTTATGCTTGTCTTCTAG-3′; H1p, Cy5-5′-AATGTAACAGTAACACACTCTGTTAACC-3′-BHQ, with the primer concentrations both at 0.4 μM and the probe at 0.12 μM. An additional set of hemagglutinin primers and probe for H3 seasonal influenza virus was also included, namely, AH3F, 5′-CCTTTTTGTTGAACGCAGCAA-3′, AH3R, 5′-CGGATGAGGCAACTAGTGACCTA-3′, and H3p, VIC-5′-CCTACAGCAACTGTTACC-3′-MGBNFQ, and the primers and probe concentrations were again at 0.4 μM and 0.12 μM, respectively. The generic influenza A virus matrix probe (VIC-5′-TCYTGTCACCTCTGAC-3′-MGBNFQ) was replaced with another generic probe, namely, FAM-5′-CCCCTCAAAGCCGA-3′-MGBNFQ, and used at the same concentration (0.16 μM) along with the AMF primer (0.4 μM) and AMR primer (0.8 μM) as previously reported (25). The reporter label (Cy5) on the influenza B virus probe (BNP probe) was converted to Quasar 705, by accessing the fifth channel (crimson) on the Rotorgene 6000 instrument and creating a pentaplex assay. The concentrations of primers (BNP-F and BNP-R) and the Quasar 705 probe for the influenza virus B component of the assay were, however, increased to 0.2 μM and 0.08 μM, respectively. The MS2 bacteriophage internal control (IC) component was identical to that described previously (25), with the primers (MS2 F1 and MS2 R1) and ROX-labeled MS2 probe concentrations each at 0.08 μM. The pentaplex assay was performed with the use of the SuperScript III Platinum one-step qRT-PCR enzyme (Invitrogen, Paisley, United Kingdom) in a reaction volume of 25 μl (containing 3 mM MgSO_4_) and the Rotor-Gene 6000 instrument. The amplification conditions were incubation at 50°C for 30 min and at 95°C for 2 min, followed by 45 cycles of denaturation at 95°C for 15 s and annealing and extension at 60°C for 1 min. Fluorescence was measured for each of the five channels at the end of each cycle. The upgraded pentaplex assay was then subjected to a program of validation to assess its performance before allowing it to supersede our quadriplex assay (25) as our laboratory's front-line diagnostic test. Briefly, 125 routine clinical respiratory specimens, comprising 50 influenza virus A-positive (38 H3N2 and 12 H1N1pdm09), 10 influenza B virus-positive, and 65 negative specimens were processed by both real-time assays in parallel. Concordant results were obtained with both assays for the influenza A and influenza B virus-positive specimens and negative specimens, with the pentaplex assay providing additional typing data for influenza A virus positives, i.e., distinguishing (H1N1)pdm09 from H3N2 isolates. The *C_T_* values for the comparable components (generic influenza A and B viruses) of both assays were broadly similar. However, the 12 A(H1N1)pdm09 samples gave a consistently lower *C_T_* value, between 1 and 1.5, with the pentaplex assay. The performance of both assays was also assessed using a number of external quality assessment (EQA) panels: the QCMD 2011 influenza hemagglutinin typing EQA program (14 samples) and the QCMD 2011 influenza A and B RNA EQA program (12 samples), obtained from Quality Control for Molecular Diagnostics (www.qcmd.org). The annual HPA Influenza Molecular Proficiency Panel 7 (for 2012), containing 14 samples, was also subjected to both assays in parallel. Both assays delivered a 100% score with the three EQA panels, giving concordant results for the common influenza A and B virus components. The quadriplex assay was, however, able to type the H5 samples, while the pentaplex assay could distinguish and type the H1N1pdm09 and H3N2 samples in the panels, in line with their defining assay attributes. Serial 10-fold dilutions of RNA extracted from virus stocks of A/Solomon Island/3/2006 (H1N1), A/Wisconsin/67/2005 (H3N2), and B/Panama/45/90 were analyzed by both real-time assays, and their limits of detection were determined to be identical. The analytical sensitivity of the pentaplex assay was calculated using serial dilutions of plasmid constructs containing the matrix gene of A/PR/8/34 (PR8) and the nucleoprotein gene target site of B/Florida/4/2006, and it was found to be ∼5 and 15 genome equivalents per PCR for influenza A and B viruses, respectively.

### (ii) WIV-ISP.

An in-house duplex qRT PCR was developed for the simultaneous detection of influenza A and B viruses. The qRT-PCR was performed using the SuperScript III Platinum one-step qRT-PCR kit (Invitrogen) on the Mx3005p QPCR system (Agilent Technologies, Stockport, United Kingdom). Primers and probes for influenza A virus target the matrix gene (27), and those for influenza B virus target the HA gene (28). The qRT-PCR mixture was prepared in a reaction volume of 25 μl containing 0.8 μM influenza A virus forward and reverse primers (InfA For and InfA Rev), 1 μM influenza B virus forward and reverse primers (InflB For and InflB Rev), 0.2 μM influenza A virus probe (InfA Probe FAM-BHQ1), 0.1 μM influenza B virus probe (InflB HEX-BHQ1), and 0.5 μl of SuperScript III RT/Platinum *Taq* mix. The cycling conditions started with an initial incubation at 50°C for 30 min and 95°C for 2 min, followed by 45 cycles of denaturation at 95°C for 15 s and an extension step at 55°C for 30 s. The fluorescence signal was acquired at the end of each cycle. This assay was developed for use in the WIV-ISP, which oversees the influenza surveillance system in Belgium (29). The quality of the assay is controlled through regular internal and external quality assessment programs, and the clinical data collected with this assay are reported to the WHO/Europe Influenza Surveillance Network (EuroFlu.org) for epidemiological monitoring. The WIV-ISP assay was therefore chosen as the reference test for the archived samples.

### Primers and probes of the SAMBA Flu duplex test.

SAMBA primers and probes targeting influenza virus conserved regions (matrix protein gene of influenza A virus and nonstructural protein gene of influenza B virus) were designed by first analyzing available sequences by segment from the Influenza Virus Resource (National Center for Biotechnology Information; http://www.ncbi.nlm.nih.gov/genomes/FLU/FLU.html). Sequences of each gene segment were aligned with the multiple alignment using the fast Fourier transform (MAFFT) program, available from the European Molecular Biology Laboratory—European Bioinformatics Institute (EMBL-EBI; http://www.ebi.ac.uk/Tools/msa/mafft/). Conserved regions suitable for SAMBA were identified by the Jalview program (version 2.3; University of Dundee, Dundee, United Kingdom). Primers and probes targeting influenza A or B virus conserved regions were designed by using Primer3 (version 0.4.0; http://frodo.wi.mit.edu/) followed by minor modifications for compatibility with SAMBA. These oligonucleotides were further analyzed *in silico* for specificity by using the Basic Local Alignment Search Tool (BLAST) and for predicted secondary structure, G/C content, and potential hetero- and homodimer formation with the use of OligoAnalyzer (version 3.1; Integrated DNA Technologies). The same primer-probe sets were adapted for RT-PCR and qRT-PCR used in production of *in vitro* transcripts and quantification of cultured stock (described above).

Through the analysis of influenza A/B virus sequences, the influenza A virus matrix protein (M) gene and influenza B virus nonstructural protein (NS) gene were identified as the most suitable targets for SAMBA Flu duplex test due to their high degree of sequence conservation. Conserved regions with optimal target length and G/C content were chosen for further design of amplification primer-probe sets. Coincidentally, the initial design of SAMBA Flu A primers overlapped with the CDC universal influenza A virus primers by 63.6% (forward) and 75% (reverse) (27). The CDC influenza A virus primers were therefore modified and adapted for SAMBA as one of the tentative primer sets for evaluation. This primer set (A-F and A-R), which amplified a 106-nucleotide (nt) target region of the influenza A virus M gene, was eventually selected. Influenza B virus primer set (B-F and B-R) was designed to target a 109-nt conserved region of the influenza B virus NS gene, whereas the IC primer set (IC-F and IC-R) amplifies a 102-nt region of the MS2 phage genome, which showed no sequence similarity with known human pathogens. The T7 promoter with a linker sequence (5′-AATTCTAATACGACTCACTATAGGGAGAAGG-3′) was added to the 5′ end of these three reverse primers. The detector probe, which was labeled with multiple hapten moieties, and capture probe were designed for each target region to recognize sequences on the single-stranded SAMBA product. Table 1 lists the oligonucleotides designed for the SAMBA Flu duplex test.

**Table 1:** Sequences of primers and probes used in the SAMBA flu duplex test

Target	Oligonucleotide	Sequence (5′–3′)
Influenza A virus M gene	A-F	AGGGCATTYTGGACAAAKCGTCTA
	A-R^*a*^	GACCRATYYTGTCACCTCTGAC
	A-Det	CGGTGAGCGTGAACACAAAT
	A-Cap	CCTAAAATCCCCTTAGTCAG
Influenza B virus NS gene	B-F	CATCTTCTTCATCCTCCACTGTAA
	B-R^*a*^	TAYAAGTCCTTATYAACTCTGCATA
	B-Det	CCACTCTGGTCATATGCATT
	B-Cap	CAGTAGCAACAAGTTTAGCA
MS2 phage	IC-F	CTCGCGTTCACAGGCTTACA
	IC-R^*a*^	TGGGTTGCCACTTTAGGCAC
	IC-Det	AATACACCATCAAAGTCGAG
	IC-Cap	TGTAGCGTTCGTCAGAGCTC

a T7 promoter and a linker sequence, 5′-AATTCTAATACGACTCACTATAGGGAGAAGG-3′, was attached to the 5′ end of the reverse primers. F, sense primer; R, antisense primer; Det, detector probe (labeled with multiple hapten moieties); Cap, capture probe lined on dipstick; M, matrix protein gene; NS, nonstructural protein gene.

### Detection of influenza A/B viruses with the SAMBA Flu duplex test.

**Fig 1:**
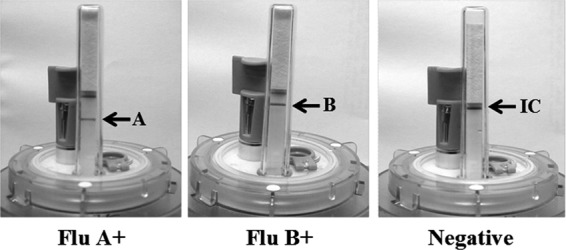
The SAMBA Flu duplex test. Examples of influenza virus A-positive, influenza virus B-positive, and negative results. The signal strengths of the IC and influenza A/B test lines were graded from 0 to 5 according to an in-house scoring chart. A blank dipstick (no IC or influenza A/B signal) indicated an invalid test run and required repeat of the entire test procedure. The presence of the IC signal is not required for a valid influenza virus-positive call, i.e., the IC can be out-competed by a very high viral load sample in rare cases.

Specimens received at the University of Cambridge were prepared in three aliquots for SAMBA testing, discrepant analysis, and repeat testing when necessary. A 250-μl aliquot of the swab sample was extracted using the SAMBAprep machine and proprietary lysis, wash, and elution buffers as described previously (24). To the lysis buffer was added 1,500 PFU of phage (the optimal input was titrated for each lot of phage culture to maintain an efficient triplex amplification of influenza A/B virus target and phage control) diluted in UTM as the internal control. The isothermal amplification and detection steps were performed in the enclosed SAMBAamp system (Fig. 1). The detection of amplification products was performed as previously described, with the exception that the process was performed in the enclosed device (30). For data analysis, the dipstick signal was scored from 0 to 5, with 0 being negative and 5 being strong positive, according to the in-house scoring chart (26). The total turnaround time was 135 min.

### Statistical analysis.

The Pearson product-moment correlation coefficient (*r*) was used to compare the *C_T_* value of the qRT-PCR product and the signal strengths of the SAMBA tests. The 95% confidence intervals (CIs) were calculated using VassarStats (http://vassarstats.net/).

## RESULTS

### Analytical performance. (i) Analytical sensitivity.

The limit of detection (LoD) of the SAMBA Flu duplex test was determined by testing dilutions of quantified cultured virus (the genome copy number, by using the in-house qRT-PCR) spiked in pooled negative swab specimens. The LoD was defined as the lowest concentration at which 95% of 20 replicates tested positive. A/Perth/16/2009 (H3N2) and B/Brisbane/60/2008 were used as the representative strains for influenza A and B viruses, respectively. First, 1,000, 500, 250, 200, 150, and 75 copies of influenza A/B viruses were tested in quadruplicate. All dilutions except that with 75 copies yielded 100% positivity. Concentrations above 75 copies were subsequently tested in increments of 5 copies in 20 replicates until at least 95% of the replicates tested positive. According to this procedure, the LoD of the SAMBA Flu duplex test was determined to be 95 copies/test and 85 copies/test for influenza A and B viruses, respectively.

### (ii) Analytical specificity.

A panel of common respiratory pathogen culture supernatants (Table 2) was used to validate the analytical specificity. All viruses tested negative by SAMBA.

**Table 2:** Culture panels used in analytical studies

Respiratory pathogen	Type, subtype, or strain	Result
Analytical specificity panel		
Adenovirus	5	NEG
Adenovirus	7	NEG
Coronavirus	QC43	NEG
Coronavirus	229E	NEG
Coxsackievirus	A9	NEG
Coxsackievirus	B5	NEG
Echovirus	6	NEG
Measles virus^*a*^	NA^*c*^	NEG
Mumps virus^*a*^	NA	NEG
Parainfluenza virus	1	NEG
Parainfluenza virus	2	NEG
Parainfluenza virus	3	NEG
Parainfluenza virus	4	NEG
Parechovirus^*a*^	NA	NEG
Respiratory syncytial virus	A	NEG
Respiratory syncytial virus	B	NEG
Rhinovirus	72	NEG
*Bordetella parapertussis*	NA	NEG
Escherichia coli	NA	NEG
Haemophilus influenzae	NA	NEG
Neisseria meningitidis	NA	NEG
Staphylococcus aureus	NA	NEG
Streptococcus pneumoniae	NA	NEG
Analytical reactivity (inclusivity) panel of influenza viruses		
A/New Caledonia/20/99	H1N1	+ (4)
A/Brisbane/59/2007	H1N1	+ (5)
A/England/195/2009	A(H1N1)pdm09	+ (5)
A/Wisconsin/67/2005	H3N2	+ (5)
A/Brisbane/10/2007	H3N2	+ (4)
A/Perth/16/2009	H3N2	+ (5)
A/turkey/Italy/3889/1999	H7N1	+ (5)
Av/1306/2007	H7N2	+ (4)
A/Teal/England/2006^*b*^	H5N3	+ (4)
B/Victoria/504/00	NA	+ (5)
B/Guangdong/120/00	NA	+ (5)
B/Hawaii/10/01	NA	+ (5)
B/Brisbane/32/2002	NA	+ (5)
B/Malaysia/2506/2004	NA	+ (5)
B/Brisbane/3/2007	NA	+ (3.5)
B/Brisbane/60/2008	NA	+ (5)
B/Wisconsin/1/2010	NA	+ (5)
A/New Caledonia/20/99	H1N1	+ (4)
A/Brisbane/59/2007	H1N1	+ (5)
A/England/195/2009	A(H1N1)pdm09	+ (5)
A/Wisconsin/67/2005	H3N2	+ (5)

a Confirmed-positive clinical isolates were tested.

b For this virus, 45 PFU/test was assayed. All other panel members were tested at 200 copies/test (determined via the in-house qRT-PCR).

c NA, not applicable.

### (iii) Analytical reactivity (inclusivity).

A panel comprising 18 influenza viruses, including 3 H1N1, 4 H3N2, 1 each of H5N3, H7N1, and H7N2 subtypes, and 8 influenza B viruses were tested to determine the detection spectrum of the SAMBA Flu duplex test. The copy numbers of these cultures were quantified by using the in-house qRT-PCR assay, except for A/Teal/England/2006 (quantified in PFU), owing to its low sample volume. Two hundred copies/test of the panel member and 45 PFU/test of A/Teal/England/2006 were tested in duplicate, and all tested positive (Table 2).

### (iv) Dynamic range of the IC.

The dynamic range of the IC was analyzed by testing 10-fold serial dilutions of the cultured virus in pooled clinical samples from the highest possible concentration (40.74 and 36.47 million copies/test for influenza A and B viruses, respectively). The IC signal remained visible at 4.074 and 3.647 million copies/test of influenza A and B virus, respectively, and was out-competed at higher levels of influenza virus. In addition, the potential competitive inhibition of the assay was evaluated by using samples that simulated coinfection with various concentrations of both influenza A and B viruses in a single reaction mixture. Five hundred copies of influenza A virus were mixed with 500, 5,000, 50,000, and 500,000, copies of influenza B virus, and vice versa. These simulated coinfection samples were tested in duplicate. Successful detection of both viruses was observed when two viral concentrations were within a 100-fold difference, e.g., 500 and 50,000 copies. At a 1,000-fold difference in concentrations, the virus with the lower level was out-competed. The internal control line was visible at all simulated concentrations.

### (v) Reproducibility.

An in-house quality control (QC) panel consisting of negative, medium-positive (200 copies/test), and low-positive (100 copies/test) samples was used to validate the performance of production lots of reagents throughout this project and to analyze test reproducibility. Each panel was composed of four replicates of each panel member (influenza A virus medium positive, influenza A virus low positive, influenza B virus medium positive, influenza B virus low positive, and negative [20 samples in total]) and was tested by three operators to evaluate interoperator reproducibility. Additionally, the panel testing was performed by one operator on three different days to establish interday reproducibility. All replicates tested by each operator as well as the overall results (100 replicates in total) met the internal QC criteria (0% positivity for negatives, 100% positivity for medium positives, and at least 50% positivity for low positives).

### Clinical evaluation of the SAMBA Flu duplex test.

The clinical evaluation of the SAMBA Flu duplex test was conducted by testing samples from two public health laboratories: HPA Cambridge, United Kingdom, and WIV-ISP Brussels, Belgium. The details of the clinical samples are described in the previous section. In brief, 41 prospective nasal/throat swab samples were collected during the 2011-2012 winter season from HPA Cambridge and were tested fresh (Table 3). A further 287 archived nasopharyngeal swab specimens were provided by WIV-ISP Brussels (Table 3). The routine qRT-PCR assays used by these two collaborating centers were used as the comparator tests. The comparator test results were initially blinded, and the SAMBA results were sent to and compared with the reference tests by the collaborators at the end of the evaluation.

**Table 3:** Clinical evaluation of the SAMBA Flu duplex test with qRT-PCR as the comparator method^*a*^

Test	No. of specimens					% sensitivity (95% CI)	% specificity (95% CI)	% PPV (95% CI)	% NPV (95% CI)
	Total	Q^+^ S^+^	Q^+^ S^−^	Q^−^ S^+^	Q^−^ S^−^				
Prospective samples									
Influenza A virus	41	11	0	2	28	100 (74.1–100)	93.3 (78.7–98.2)	84.62 (57.8–95.7)	100 (87.9–100)
Influenza B virus	41	1	0	0	40	100 (20.7–100)	100 (91.2–100)	100 (20.7–100)	100 (91.2–100)
Archived samples									
Influenza A virus	287	124	0	2	161	100 (97.0–100)	98.8 (95.6–99.7)	98.4 (94.4–99.6)	100 (97.7–100)
Influenza B virus	287	93	0	0	194	100 (96.0–100)	100 (98.1–100)	100 (96.0–100)	100 (98.1–100)
Overall performance									
Influenza A virus	328	135	0	4	189	100 (97.2–100)	97.9 (94.8–99.2)	97.1 (92.8–98.9)	100 (98–100)
Influenza B virus	328	94	0	0	234	100 (96.1–100)	100 (98.4–100)	100 (96.1–100)	100 (98.4–100)

a Q, qRT-PCR result; S, SAMBA result; CI, confidence interval; PPV, positive predictive value; NPV, negative predictive value.

In the initial testing, the SAMBA Flu duplex test detected 135 influenza A-positive and 94 influenza B-positive concordant samples. Four samples tested influenza A positive by SAMBA but negative by qRT-PCR, and two samples tested negative by SAMBA but influenza B positive by qRT-PCR (*C_T_* values of 37.97 and 37.86). To resolve the discrepancy and to prevent any testing bias, these six discordant samples were blind tested alongside 10% of the concordant ones (randomly selected; 38 in total), using two-step typing/subtyping real-time RT-PCR assays for influenza A(H3), A(H1N1)pdm09, former seasonal H1N1, and influenza B viruses routinely used for influenza surveillance at PHE Colindale. The 32 concordant samples gave the same results as SAMBA (and the comparator qRT-PCR) by the PHE real-time assays. Four influenza A virus SAMBA-positive qRT-PCR-negative samples tested negative, and two influenza B virus SAMBA-negative qRT-PCR-positive samples also tested negative. In summary, through the analysis of 328 clinical specimens, the SAMBA showed a resolved clinical sensitivity and specificity of 100% and 97.9% for its influenza A virus test and a sensitivity and specificity of 100% and 100% for its influenza B virus test (Table 3). The performance between the prospective study, although small in sample size, and the retrospective study was similar. Notably, out of these test, only one sample from Belgium initially gave an invalid result. Upon repeat, this sample tested negative (concordant). This gave an invalid rate of 0.3%. The SAMBA Flu duplex test detected almost all the positive cases (97.4% [227/233]) with a clearly visible signal (≥3), even in samples with low viral load (*C_T_* ≥ 30). In the six samples (2.6%) showing a low dipstick signal, two had a very low viral load (SAMBA ID 52, with SAMBA signal of 1, *C_T_* of 37.05, and SAMBA ID C4, with SAMBA signal of 1 and *C_T_* of 34.83), and the other four were later confirmed (resolved by qRT-PCR and also retested by SAMBA) to be false positives, possibly due to degradation of the viral genome in the original samples or contamination during the extraction process. A weak negative correlation was observed between the signal strength of the SAMBA test and the *C_T_* values from the qRT-PCR assays (*r* = −0.35; *P* < 0.0001).

## DISCUSSION

The evolving genetic and antigenic variabilities of influenza viruses are the main obstacle in managing influenza epidemics and pandemics. Rapid and accurate diagnostic testing close to the source is important for the control of outbreaks and detection of emerging influenza viruses. The correlation between the efficacy of disease control and early initiation of medical intervention accentuates the need for an improved influenza virus diagnostic assay that offers the ease-of-use of a rapid immunoassay and the accuracy of molecular technology. Recently, there have been a number of commercial molecular tests, such as the IQuum Liat Influenza A/B assay, the Simplexa Flu A/B, and RSV Direct and Cepheid Xpert Flu test, designed for use in near-patient settings (31–34). These assays simplify the technical manual steps of molecular testing by use of an automated device. Nevertheless, due to the use of real-time PCR technology, these assays require specialized equipment and software for sample processing and data interpretation. The SAMBA Flu duplex test described in this study utilizes isothermal amplification and visual dipstick detection of viral nucleic acid (amplification product) to further reduce the complexity of molecular testing. An internal control based on bacteriophage MS2 was also integrated into the test to monitor the entire test procedure, from extraction to detection. Analytically, the assay was able to detect 95 and 85 copies/test (380 and 340 copies/ml) of influenza A and B viruses, respectively. The SAMBA also demonstrated a promising clinical performance that was comparable to the routine qRT-PCR used in two national reference laboratories (Table 3). In addition, the test was shown to be analytically specific against common respiratory pathogens and was found to be reactive to over 20 different subtypes/strains of influenza A/B viruses. In the rare event of influenza A and B virus coinfection, the SAMBA Flu duplex test was capable of detecting both viruses when their viral loads were within a 100-fold difference of each other.

Evaluation of a new assay in a blinded manner is directly linked to the validity of the claimed test performance. Indeed, a significant quality concern over rapid tests for influenza virus is their failure to report whether the clinical evaluation was conducted in a blinded fashion (35). For the clinical evaluation of the SAMBA Flu duplex tests, the clinical samples were tested blind, including the testing of archived samples from WIV-ISP, Belgium. During the 2011-2012 winter in England, influenza virus activity was low, and the H3N2 subtype (A/Perth/16/2009-like virus) was the dominant strain, out-competing both the A(H1N1)pdm09 and influenza B viruses. For this reason, retrospective archived swab samples, collected by WIV-ISP, Belgium, from 2010 to 2012 (including between-season months), were used to supplement the small number of prospectively collected samples obtained in Cambridge. This ensured that the clinical evaluation of the SAMBA Flu duplex test was not limited to the diagnosis of the H3N2 subtype. In total, the SAMBA test showed promising performance (>97% sensitivity and specificity for influenza A and B viruses detection compared to the routine qRT-PCR used by two national reference laboratories) in this population of clinical samples. The invalid rate (no IC and test signal and required repeat of the entire test procedure) was found to be 0.3% (1/328). As a comparison, invalid testing due to failure of the internal control was reported to be 5.7% (of 192 samples) for the Prodesse ProFlu+ assay, and 3.5% (of 202 samples) was reported for the for xTAG RVP test (36, 37).

The novelty of an influenza virus rapid test is its feature allowing the test result to be interpreted by eye on the dipstick. Such visual detection, intended to improve the usability in near-patient diagnosis, might result in diminished test sensitivity if the dipstick signal for a positive sample is not easily distinguishable from that for negative cases by the operator. Stevenson and Loeffelholz reported weak dipstick signal intensity in nearly 43% of the positive pandemic A(H1N1)pdm09 samples tested using the QuickVue Influenza A+B test (38). The large proportion of weak positive results could adversely affect the overall test performance due to operator-dependent error in identifying the weak signal as positive. The principle of the SAMBA test takes advantage of the explosive accumulation (10^9^-fold increase in 1 to 2 h) of single-stranded amplification product by isothermal amplification in conjunction with the finely adjusted amplification/detection formulation and high yield of viral RNA purification, to achieve an “all-or-none” signal profile, i.e., a positive test signal was almost always visibly strong (≥3 signal) (39). This all-or-none feature (reflected in the weak correlation between the dipstick signal and *C_T_* value, *r* = −0.35 and *P* < 0.0001) of the SAMBA Flu duplex test greatly minimized the ambiguity of test interpretation. As for the IC signal of the SAMBA Flu duplex test, all negative samples gave a strong IC signal (>4), although only 29.2% (68/233) of the positive samples gave an IC signal of ≥3. The lower IC signal for the positives could be related to the assay being optimized in favor of the amplification of the influenza virus targets (in order to achieve high test sensitivity). This could be remedied by retitration of input of the IC at the extraction step and/or the IC primer in the amplification mix. Nevertheless, the IC system developed here was effective in indicating true-negative results by giving strong IC signals, and the presence of the IC signal is not required for strong influenza virus-positive cases (a separate negative run was always included to monitor the amplification of the IC).

In terms of the turnaround time, the SAMBA Flu duplex test currently takes 2 h and 15 min for the entire test procedure. Compared to other commercial molecular tests that do not have an integrated extraction procedure (which can take 3 to 4 h from extraction to amplification/detection), the SAMBA maintains a competitive advantage (20–23). However, other commercial tests designed for point-of-care use have much shorter turnaround times (from 20 min to just over 1 hour) than the SAMBA (33, 34, 40). This disadvantage could be addressed by reducing the detection and amplification times, which are currently longer than required for the expected test sensitivity, with the objective to reduce the turnaround time to approximately 1.5 h. The SAMBA has a capacity to process four samples in one test run. This throughput makes the SAMBA particularly suitable in the near-patient settings, such as in physicians' clinics, where the sample number is small and a sophisticated testing procedure is not permitted. Although the SAMBA showed good sensitivity for influenza virus typing, the samples would still need to be referred to a centralized laboratory for subtype identification in the case of epidemiological monitoring.

Molecular diagnosis on a lateral/vertical flow system has been described for the detection of other viral and parasitic infections (41, 42). However, these assays lacked an integrated nucleic acid extraction protocol and have yet to demonstrate clinical efficacy in practical settings. In summary, we report here a molecular influenza virus duplex test that showed clinical performance comparable to that of the conventional qRT-PCR tests used in national reference laboratories in the United Kingdom and Belgium. Moreover, it demonstrated practical implementation of a new format of a nucleic acid amplification test that couples high sensitivity of molecular detection and a low technical requirement of data interpretation via the dipstick. The chemistry at present has been integrated into a semiautomated platform and is undergoing further development into a fully automated test. It is envisaged that the accurate and ease-of-use features of the assay will bring state-of-the-art molecular testing for the influenza virus closer to patients in the near future.
